# Insights from Tandem Mass Tag (TMT) Proteomic Analysis on Protein Network Modification in Control of Yak Hair Follicle Cycle

**DOI:** 10.3390/ijms26041532

**Published:** 2025-02-12

**Authors:** Shijie Li, Yan Cui, Sijiu Yu, Junfeng He, Rui Ma, Bo Liao, Pengfei Zhao, Pengqiang Wei, Niayaler Robert

**Affiliations:** 1 Laboratory of Animal Anatomy & Tissue Embryology, Department of Basic Veterinary Medicine, Faculty of Veterinary Medicine, Gansu Agricultural University, Lanzhou 730070, China; 107331901031@st.gsau.edu.cn (S.L.); hejf@gsau.edu.cn (J.H.); 107332001032@st.gsau.com (R.M.); liaobo199303@163.com (B.L.); 107332201037@st.gsau.edu.cn (P.Z.); weipengqiang@gsau.edu.cn (P.W.); niayaleroert@gmail.com (N.R.); 2 Gansu Province Livestock Embryo Engineering Research Center, Department of Clinical Veterinary Medicine, Faculty of Veterinary Medicine, Gansu Agricultural University, Lanzhou 730070, China; sijiuy@126.com

**Keywords:** anagen, catagen, plateau, ppard, sfrp1, telogen

## Abstract

Highland animals have unique hair growth mechanisms to allow them to adapt to harsh living environments. Compared with other species, their hair cycle growth is affected by more environmental factors. Yaks, as highland animals, have obvious periodic hair growth characteristics in a year; this biological process is regulated by numerous proteins, but the specific molecular regulatory mechanism is still unclear. Here we analyzed the histological characteristics of yak hair follicles (HFs) at each stage and conducted TMT proteomics research. The protein expression network of yak hair at each stage and the mechanism of the yak HF growth cycle were systematically explored, and the candidate proteins Sfrp1 and Ppard were verified. A total of 3176 proteins were quantifiable and 1142 differentially expressed proteins (DEPs) were obtained at five stages of the yak hair cycle. DEPs enriched in complement activation change, tissue development, lipid metabolism, WNT pathway, VEGF pathway, JAK-STAT pathway, and PPAR pathway may promote the growth of yak hair follicles, such as Serpinf1, Ppard, and Stat3. DEPs enriched in complement system, coagulation, cell adhesion, lipid metabolic process, proliferation of epidermal cells, and estrogen pathway may promote the degeneration of yak hair, such as Sfrp1, Eppk1, and Egfr. Using Protein-Protein Interaction (PPI) analysis, we found that core nodes of DEP networks in yak skin are significantly different at three critical time points in hair follicle development, and lipid metabolism proteins are common core DEP nodes during yak HF growth and degeneration. The expression of Sfrp1 and Ppard in yak hair follicles at different periods showed they are related to yak hair cycle control. This study showed that the protein regulatory network of the yak HF growth cycle is complex and dynamically changing and revealed key candidate proteins that may affect yak hair follicle development. These findings provided detailed data for further understanding of the plateau adaptation mechanism of the yak, which is of great significance to make better use of the yak livestock resources and enhance their economic value.

## 1. Introduction

The yak is a large domestic animal and occupies an important position in the plateau ecosystem. Generally, the yak lives at altitudes between 2500 and 5500 m [1,2]. The high elevation plateau is characterized by very cold temperatures (hypothermia), dramatic climate change, and strong UV radiation, which can impose strong environmental stresses on animals living there [2,3,4]. However, yaks have evolved and adapted to these harsh environmental conditions using numerous anatomical and physiological traits as well as molecular level mechanisms to equip them for life at high altitudes [2,5]. As the first barrier of the body, yak skin resists harsh environmental factors and is characterized by a thick dermis, abundant subcutaneous tissue, and well-developed hair [1,6]. The fur of the yak can be divided into two types: coarse wool and the undercoat, which are formed by primary and secondary hair follicles, respectively [1,3]. In particular, yaks have long hair under their abdomen to keep the body warm in the range of environmental temperatures from −30 °C to 25 °C [7]. According to previous studies, a complete hair growth cycle consists of anagen, catagen, and telogen [8,9,10]. Although the hair follicle growth cycle of humans, mice, and cashmere goats can be divided into three similar stages, they each have their unique characteristics in the hair follicle growth cycle [11,12]. Human hair follicles do not have synchronous growth patterns, and each follicle is independent of the others [11]. The growth cycle of goat hair follicles lasts for a year, and an important influencing factor is the length of daylight [12]. The duration of anagen, catagen, and telogen in the mouse hair follicle cycle changes after birth [13]. In comparison, the plateau living environment gives the hair of yaks more obvious periodic growth characteristics. The yaks’ fur is constantly renewing itself in a complete growth cycle [1]. This enables local herders to harvest a large amount of yak hair every year to produce exquisite textiles, thus generating huge economic benefits. This makes yak hair an important material resource. These features also make yaks an ideal animal model for studying hair follicle growth and development.

Hair follicles are a special kind of skin appendage with self-renewal ability, mainly composed of a hair shaft, dermal papilla, and hair root sheath [14]. Numerous studies have shown that hair follicles continuously undergo growth cycle of atrophy and regeneration with these transitions controlled by complex molecular regulatory mechanisms [10,14]. It is generally believed that the dermal papilla is the signal regulation center, which is a group of specially differentiated dermal-derived cells [15]. It provides the guidance signals required to activate the epithelial progenitor cells and to initiate hair follicle regeneration and regression [10]. Epithelium-derived cells such as hair matrix cells and root sheath cells proliferate as response cells to form hair shafts, and their proliferation decreases until it stops completely during catagen [14,16]. Hair follicle stem cells exist in the bulge area of the hair follicle and differentiate to other hair follicle cells as progenitor cells. These cells have massive information exchange with other parts of the hair follicle and complete the self-renewal of the hair follicle [15]. Studies have shown that the anagen of mice is about 14 days, catagen and telogen are related to the number of hair follicle cycles and the sex of mice, and the whole growth cycle lasts for about 50 days [16,17]. The growth cycle of cashmere goats consists of telogen (December–March), anagen (March–September), and catagen (September–December) [12]. For yaks, with significant changes in environmental factors due to varying altitudes and seasons, the yak’s skin and fur directly receive environmental signals, leading to a noticeable change in the thickness of the fur. This change is determined by the cyclical growth of hair follicles. The growth cycle of yak hair follicles is similar to that of cashmere goats [6]. There are studies that have distinguished the growth cycle of yak hair follicles using high-throughput sequencing clustering, but few studies have conducted high-throughput omics studies using the changes in hair morphology [3]. The yak hair follicle cycle and its underlying mechanism may be different from other species; therefore, we used the TMT proteomics method based on hair follicle morphology to reach a deeper understanding of yak hair follicle cycle.

As the executors of gene function, proteins are directly involved in the change in biological characteristics [18]. During anagen, catagen, telogen, and the transformation of hair follicles, there is a complex protein network to maintain homeostasis and regulate each stage. Proteomics is an important tool for understanding complex systems and a promising method for elucidating complex biological mechanisms. Tandem mass tag (TMT) is one of the best quantitative proteomics methods because of its high throughput, small systematic error, and powerful function [19]. This study aims to screen for differentially expressed proteins (DEPs) at critical time points of yak hair growth, regression, and resting. DEP analysis of the critical time points of yak hair follicles can help systematically understand the mechanism of periodic growth of yak hair follicles at the protein level and provide basic information for the research on the adaptive mechanism of the yak to plateau area and hair medicine.

## 2. Results

### 2.1. Yak HFs Cycle Histological Characteristics

**Figure 1:**
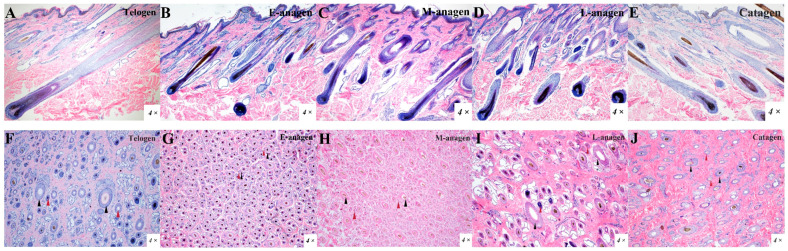
Histological structure of yak hair follicle in each stage. H&E staining of yak hair follicle on the vertical to the skin surface in each stage: (**A**) Telogen; (**B**) E-anagen; (**C**) M-anagen; (**D**) L-anagen; (**E**) Catagen. H&E staining of yak hair follicle on parallel to the skin surface in each stage: (**F**) Telogen; (**G**) E-anagen; (**H**) M-anagen; (**I**) L-anagen; (**J**) Catagen. Black arrow indicates primary hair follicle (PF). Red arrow indicates secondary hair follicle (SF).

**Figure 2:**
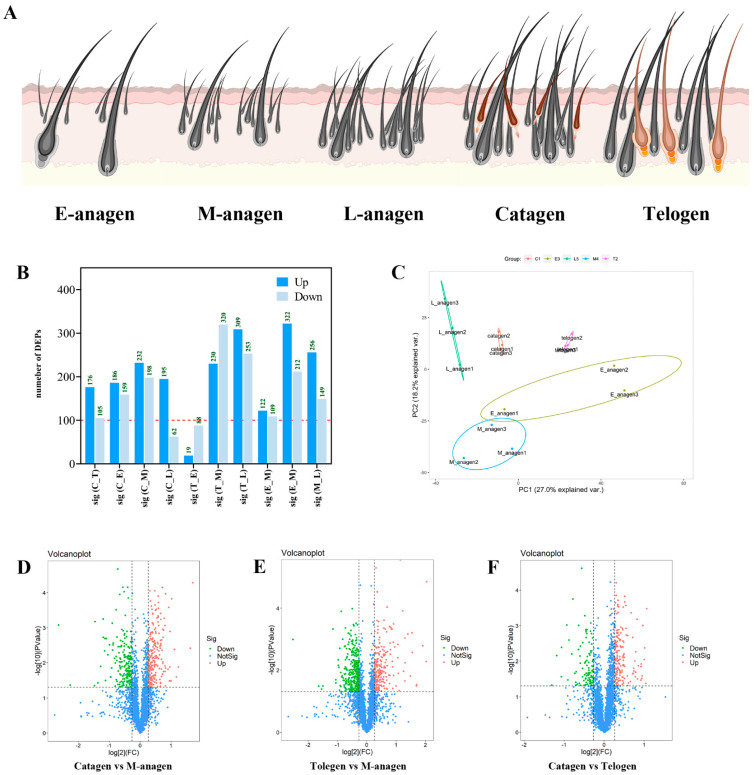
Characteristics of the cyclic growth of yak hair follicles and protein expression analysis of yak hair follicle growth in each stage. (**A**) Schematic diagram of periodic growth of yak hair follicle. (**B**) Number of DEPs between each stage of yak hair follicle growth. (**C**) PCA analysis based on the expression of proteins. (**D**–**F**) Volcano plot of DEPs at the time point of yak hair degeneration, hair growth, and hair resting.

The histological structure of yak hair follicles in telogen (January), early anagen (April), mid-anagen (June), late anagen (September), and catagen (November) were displayed (Figure 1A–J). Results showed that hair follicles grew in clusters, including primary hair follicles (coarse wool) and secondary hair follicles (undercoat). Secondary hair follicles surround primary hair follicles to form hair follicle clusters (Figure 1F–J). Secondary hair follicles begin to grow in early anagen, and primary hair follicles are the majority (Figure 1B,G). In mid-anagen, the number of secondary hair follicles increased gradually, and almost all hair follicles grew hair shafts (Figure 1C,H). At the late-anagen stage, there was little difference in size between primary hair follicles and secondary hair follicles in the same hair follicle group (Figure 1D,I). Hair follicle growth was obvious in anagen. In catagen, hair follicles began to degenerate, hair follicle density decreased, and the non-degenerative hair follicles became deeper and longer (Figure 1E,J). In telogen, the hair follicle is the largest and the hair roots are deepest in dermis, coarse wool and undercoat are the longest, and the fur is thicker than that in anagen and catagen (Figure 1A,F). When entering the next anagen, the new hair shafts then push out the old ones and a new hair cycle begins (Figure 2A).

### 2.2. Overview of TMT Proteomics Data

To explore the key protein regulatory networks of yak hair follicles cycling, skin samples from five different periods were sequenced by TMT proteomics. LC-MS/MS identified 312,570 secondary spectra. A total of 55,016 available effective spectra were obtained. A total of 32,262 peptides were identified as specific and 3176 proteins were quantifiable. The data were uploaded to the Uniprot database *Bos taurus* (37,513, 25 May 2021), and a total of 2582 annotated proteins and 1142 differentially expressed proteins were obtained (*p* < 0.05; FC ≥ 1.2). To obtain DEPs related to hair follicle growth, three time points of hair growth, degeneration, and resting were selected for further analysis. At the beginning of hair growth, the numbers of DEPs in E-anagen vs. telogen and M-anagen vs. telogen were 107 and 550, respectively (Figure 2B). Compared with telogen, 88 proteins were up-regulated and 19 proteins were down-regulated in E-anagen (Figure 2B, Figures S1A and S2C). A total of 320 proteins were up-regulated and 230 proteins were down-regulated in M-anagen (Figure 2B,E and Figure S1B). At the time point of hair degeneration, the numbers of DEPs in M-anagen vs. catagen and L-anagen vs. catage were 430 and 257, respectively (Figure 2B). Compared with catagen, 198 proteins were up-regulated and 232 proteins were down-regulated in M-anagen (Figure 2B,D and Figure S1C). A total of 62 proteins were up-regulated and 195 proteins were down-regulated in L-anagen (Figure 2B, Figures S1D and S2D). At the hair resting time point, the number of DEPs in catagen vs. telogen was 281 (Figure 2B). A total of 105 proteins were up-regulated and 176 proteins were down-regulated in catagen (Figure 2B,F). Evaluation of quantitative proteome reproducibility was performed using PCA, showing that the biological replicates were statistically consistent (Figure 2C). Obviously, there are more DEPs in M-anagen compared to telogen and catagen (Figure 2B). The protein network in E-anagen is similar to telogen, and the protein network in L-anagen is also similar to catagen. This shows that the protein regulatory network of hair follicles is becoming more complex in the process of transition between growth and degeneration. Comprehensive analysis of the two groups of DEPs at the time of hair growth and degeneration could more comprehensively reveal the key proteins of hair follicle growth and degeneration.

### 2.3. GO and KEGG Enrichment Analyses of DEGs

**Figure 3:**
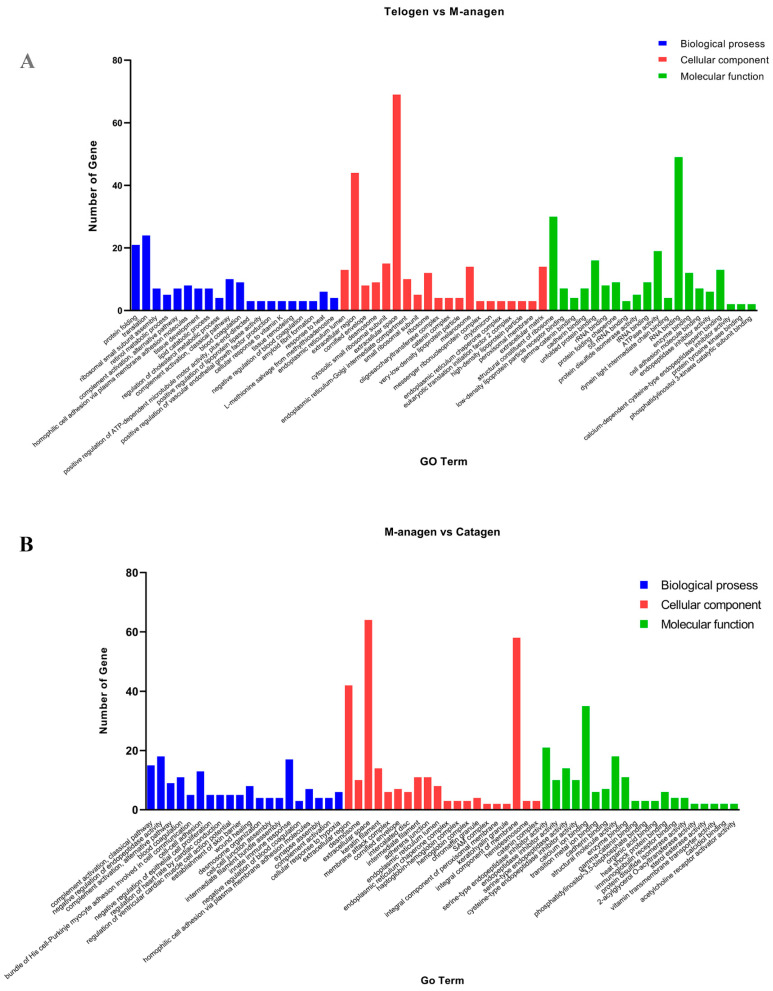
GO enrichment analysis. (**A**) Top20 GO enrichment analyses of DEPs at yak hair growth time point. (**B**) Top20 GO enrichment analyses of DEPs at yak hair degeneration time point.

**Figure 4:**
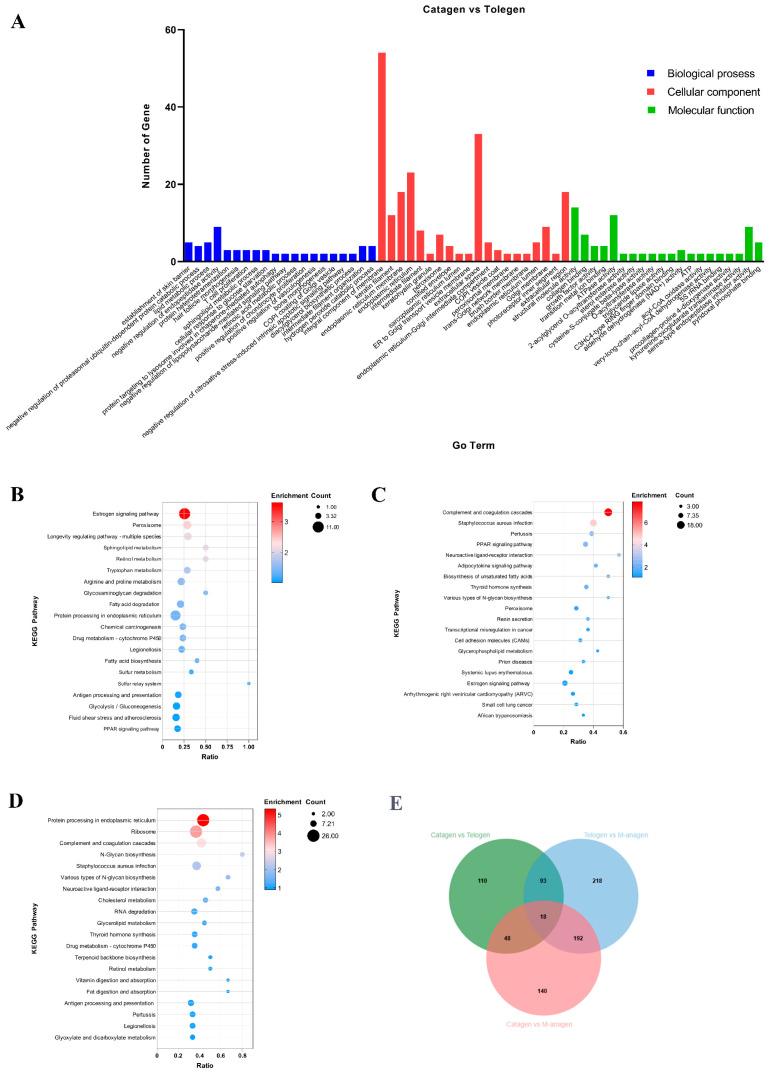
GO and KEGG enrichment analysis of DEPs at three critical time points in hair follicle growth transformation. (**A**) Top20 GO enrichment analyses of DEPs at yak hair resting time point. (**B**) Top20 KEGG pathways enrichment analyses of DEPs at yak hair resting time point. (**C**) Top20 KEGG pathways enrichment analyses of DEPs at yak hair degeneration time point. (**D**) Top20 KEGG pathways enrichment analyses of DEPs at yak hair growth time point. (**E**) Venn diagram of three time points of yak hair growth, degeneration and resting.

To understand the biological function of DEGs at the three critical time points in hair follicle development transformation, we performed GO function (*p* < 0.05) and KEGG pathway enrichment analyses (Figure 3 and Figure 4, Tables S1 and S2). GO enrichment results showed that in Biological Process (BP) classification, the main GO terms enriched in the transition of telogen to anagen were protein folding, retinol metabolism, complement activation change, tissue development and lipid metabolism (Figure 3A and Figure S2A). During the transition from anagen to catagen, the main GO terms enriched are the complement system, coagulation, cell adhesion, proliferation of epidermal cells, and formation of skin barrier (Figure 3B and Figure S2B). During the transition from catagen to telogen, the main GO terms enriched are the establishment of skin barrier, lipid metabolism, hair follicle morphology, retinoic acid metabolism, and intermediate filament tissue (Figure 4A). In Cellular Component (CC) classification, keratin filament, intermediate filament, keratinous granules, and fatty granules were significantly enriched. In Molecular Function (MF) classification, cell adhesion, protein folding, and adipokine binding were significantly enriched (Figure 3, Figure 4A and Figure S2A,B). Keratin filament and intermediate filament GO terms significantly enriched which is related to HF cycling change. The tissue development and keratinous granules GO terms are related to hair follicle regeneration. Cell adhesion, proliferation of epidermal cells, and formation of skin barrier GO terms participate in hair shaft formation. Additionally, a large number of lipid metabolism-related GO terms were significantly enriched at the three time points, indicating that lipid decomposition or accumulation may play an important role in HF cycling (Figure 3 and Figure 4A).

KEGG enrichment analysis showed that Estrogen, WNT, VEGF, MAPK, and JAK-STAT pathways related to HF development were significantly enriched (Figure 4B–D, Table S2). The estrogen signaling pathway was closely related to hair loss [20]. The WNT signaling pathway is one of the essential pathways for hair follicle formation and maintenance in the embryonic stage [21]. VEGF signaling pathway is closely related to stimulating the growth of hair follicle dermal papilla cells and maintaining hair shaft growth [22]. JAK-STAT signaling pathway is associated with the differentiation of cells in hair follicles and maintenance of hair follicle progenitor cell numbers [23]. In addition, we noticed that the PPAR pathway was significantly enriched at the three time points (Figure 4B–D). It is the most important pathway that affects downstream lipid accumulation and decomposition. In addition to disease pathways, fatty acid biosynthesis, fat digestion and absorption, cholesterol metabolism, and triglyceride metabolism signaling pathways were significantly enriched (Figure 4B–D). Significant enrichment of lipid metabolism pathways suggests that it may affect the HF cycling in yaks.

### 2.4. PPI Network Construction and Analysis

**Figure 5:**
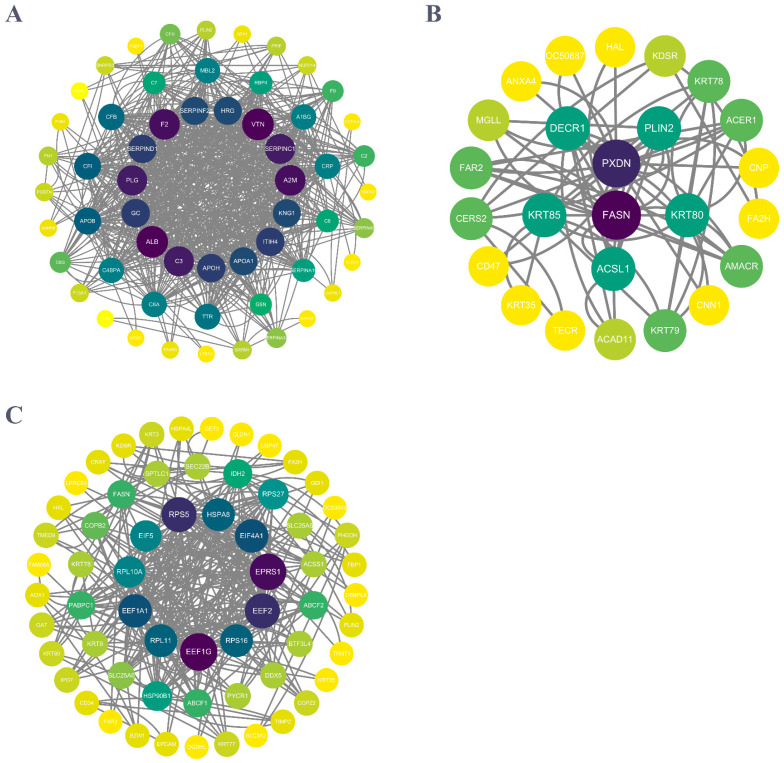
PPI network analysis protein expression changes of yak hair cycling. (**A**) PPI network of top100 DEPs at the time point of yak hair growth. (**B**) PPI network of top100 DEPs at the time point of yak hair degeneration. (**C**) PPI network of top100 DEPs at the time point of yak hair resting. Size and color of nodes are based on the extent of interprotein connectivity and *p*-value. Larger nodes have a highly connective degree. Deeper nodes are more significant.

**Figure 6:**
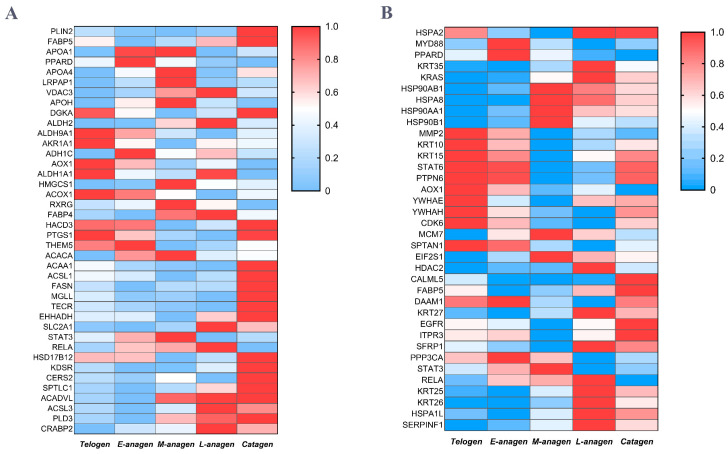
Candidate DEPs related yak hair cycle expression pattern. (**A**) Heat map of the candidate DEPs enriched in lipid metabolism expression. (**B**) Heat map of the candidate DEPs enriched in KEGG pathway-related yak hair growth expression.

PPI network analysis was performed on the top 100 differential proteins (*p* < 0.05) at the three critical time points of HF growth using cytoscape_3.9.1 software. The results showed that when the HF transitions from telogen to anagen, the core DEP nodes are composed of three parts. They are coagulation function-related proteins (Serpind1, Kng1, Serpinc1, Hrg, F2, and plasmin) and their inhibitors (Plg, Serpinf2, and Vtn), as well as lipid metabolism-related proteins (C3, Apoh, and Apoa1) (Figure 5A). We believe that yak HF growth is closely related to coagulation proteins, fibrinolytic proteins, and lipid metabolism proteins, but at the same time coagulation proteins and plasmin are directly related to skin wound healing, inflammation, and blood coagulation reaction. However, whether such results are related to the way the skin was cut at the time of sampling remains requires further research. During the HF transition from anagen to catagen, the core DEP nodes in the skin also involve lipid metabolism-related proteins (Fasn, Acsl1, Plin2, and Decr1), fibronectin binding-related protein (Pxdn), and keratin (Krt85 and Krt80) (Figure 5B). When HFs entered catagen, the significant expression change in keratin in skin confirmed the change in HFs growth state. Fibronectin expression changes are related to tissue remodeling. The expression change in a large number of lipid metabolism proteins proves that HFs degeneration is closely related to lipid metabolism. Lipid metabolism proteins are the core DEPs in HF growth and degeneration, so we have reason to think that the change in HF growth state is affected by lipid metabolism in skin. During the HF transition from catagen to telogen, core DEP nodes in skin are composed of ribosomal proteins (Rps5, Rps16, Rpl10a, Rpl11, Rps5), ribosome-binding proteases (Eef1g, Eef1a1, Eif5, Eef5, Eef2, Eif4a1, Eprs1), and heat shock protein Hspa8 (Figure 5C). However, at this point in time, the core protein nodes of DEPs network are obviously different, indicating that HF growth and regression are directly related to protein expression in the skin. Finally, Combining the GO and KEGG results, we summarized the expression variation in candidate DEPs in each stage of yak HF cycle (Figure 6A,B), and selected Ppard, the core factor of PPAR signaling pathway, and sfrp1, its upstream regulator, as validation proteins.

### 2.5. Protein Validation

**Figure 7:**
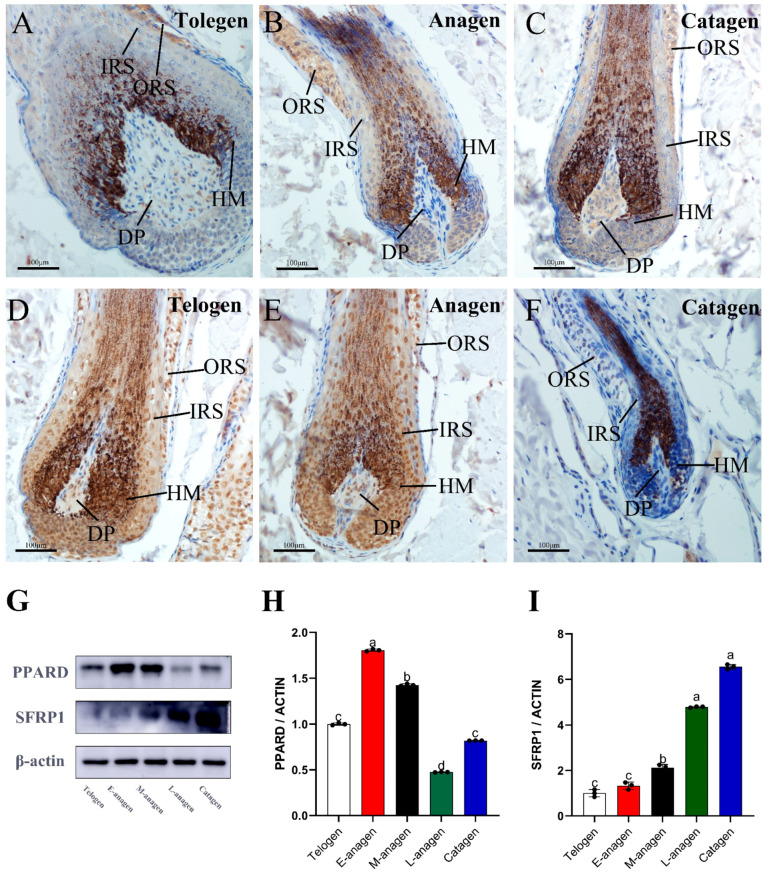
Ppard and Sfrp1 validation in yak hair cycling. (**A**–**C**) Immunohistochemical staining of Sfrp1 in telogen, anagen, and catagen of yak hair. (**D**–**F**) Immunohistochemical staining of Ppard in telogen, anagen, and catagen of yak hair. (**G**–**I**) Western blotting was used to identify the expression variation in Ppard and Sfrp1 in telogen, E-anagen, M-anagen, L-anagen, and catagen of yak skin (*n* = 3). Data are means ± SD. a, b, c, d are significantly different.

The candidate proteins Sfrp1 and Ppard enriched in the WNT and PPAR signaling pathways were verified by experiments. Immunohistochemical staining was used to verify their expression in anagen, catagen, and telogen yak hair follicles. Sfrp1 was expressed in the hair matrix, hair papilla, and outer root sheath of hair follicles (Figure 7A–C). Compared with other HF growth periods, the expression of Sfrp1 in dermal papilla (DP) of catagen was significantly enhanced (Figure 7C). Ppard was expressed in the hair matrix, dermal papilla, inner root sheath, outer root sheath, and connective tissue sheath of hair follicles (Figure 7D–F). Compared with other HF growth stages, the expression of Ppard in inner root sheath, outer root sheath and dermal papilla of catagen decreased significantly (Figure 7F). We used WB to verify their abundance in E-anagen, M-anagen, L-anagen, catagen, and telogen; β-actin was used as an internal reference, and the results were consistent with LC-MS/MS data (Figure 7G–I). Compared with other HF growth periods, the expression level of Sfrp1 was up-regulated in L-anagen and catagen, suggesting Sfrp1 is related to hair degeneration (Figure 7G,I). Ppard expression was up-regulated in E-anagen, M-anagen, and down-regulated in catagen, suggesting Ppard may play a role of activator of hair growth (Figure 7G,H).

## 3. Discussion

The hair follicle is a mini organ with the ability to renew itself. This periodic development and regression occur throughout life and complex molecular signaling networks control this process [24]. Hair follicle growth is accomplished by the cooperation of various cells in the hair follicle, involving the signal communication among the dermis, epidermis and subcutaneous tissue [15]. There are a large number of classical pathways involved in the formation of HFs during the embryonic stage and HF cycling after birth such as the WNT, BMP, TGF-β, and NOTCH signaling pathways [15]. The hair follicle growth of different species is different. For yaks, fur is especially important to maintain body temperature and resist adverse environmental factors, which makes hair growth of yak to have seasonal alternation characteristics [6,8]. In this study, the hair follicle growth period of yak was determined by the hair follicle tissue structure characteristics, while the high-throughput proteomics revealed the protein expression network at each stage of yak hair follicle and the key DEPs of hair follicle transformation time points.

The hair follicle development of yak goes through a complete cycle of anagen, catagen and telogen annually. Anagen is characterized by a large number of new secondary hair follicles occurrence, and the old telogen hair replacement. The new HFs do not appear in catagen while some HFs regress, but the HFs that have not regressed gradually penetrate into the dermis and continue to grow. At the telogen stage, hair follicles are in a state of stable growth, becoming large and penetrating deep into the lower dermis. According to the amount and classification of the DEPs, it can be seen that E-anagen and L-anagen are the transitional periods of HF growth state alteration. Hair keratin (K13, K79, K82, and K83) and inner root sheath-specific keratin (K25, K71, and K27) were up-regulated during anagen and catagen, and down-regulated during telogen, while keratin K10 and K24 mainly distributed in epidermis showed no such changes (Figure 6B) [25,26,27]. This fluctuation of keratin expression also indicates at the molecular level that hair follicle growth reaches its peak in catagen, and then regresses into telogen, confirming the rational of the study.

After analysis of DEPs associated with HFs the three time points using GO terms and KEGG pathways, we found differences in protein network expression at each stage. Specifically, tissue development, cell–cell adhesion, intermediate filament organization, and epithelial cell proliferation GO terms are closely related to hair follicle function. Lamc2, a protein associated with tissue development, is an isolaminin (Laminin–5) expressed by basal keratinocytes. It is an important protein for keratinized tissue regeneration and one of the functional proteins related to the development of hair follicles in sheep [28,29]. Pkp1, Dsp, and Dsc2 cell adhesion-related proteins are involved in the formation of desmosome and intermediate filaments. Their deletion can cause hair follicle development disorders and thinning of hair [30,31,32]. Epithelial cell proliferation-related DEPs include Sfrp1, Ppard, Eppk1, of which Eppk1 specifically interacts with keratin filaments in the epidermis [33]. Its expression was highest in catagen, which was consistent with hair keratin expression. This suggests that Eppk1 might be one of the proteins involved in hair growth. WNT, JAK-STAT, VEGF, MAPK, ESTROGEN signaling pathways are involved in the regulation of yak HF cycling growth. Previous studies have shown that activation of the WNT signaling pathway can induce human and mouse hair follicles to enter anagen, while reduction in WNT ligands or increased expression of WNT antagonists induces dysregulation of the murine hair follicle cycle and causes alopecia [34]. Down-regulation of Serpinf1 stimulates proliferation of DP cells, promotes hair follicle growth and prolongs anagen in human [35]. Sfrp1 and Serpinf1 are inhibitors of WNT signaling pathway, and their expression is increased in L-anagen and catagen, which may inhibit HF growth through the WNT signaling pathway (Figure 6B). This is consistent with the results in human and mouse. Stat3 is required for wound healing and anagen progression in the hair cycle [36], which is highly expressed in E-anagen and M-anagen of yak hair follicles and may be involve in secondary HF regeneration (Figure 6B). Kras has been reported to be associated with activating HF cycling (Figure 6B) [37]. Its expression was low at the telogen stage in the yak. Loss of Egfr in mice results in wavy hair phenotype and its over activation results in hairless skin [38].

PPI protein network analysis showed that there were significant dynamic changes in the DEPs network at the three critical time points of HF cycling, and lipid metabolism factors were the main core nodes of DEP network during the transformation of yak hair growth and degeneration. Combined with GO and KEGG results, retinoic acid metabolism, retinol metabolism, lipid metabolism-related proteins and PPAR pathways were significantly enriched at the time point of hair growth and degeneration, indicating that lipid metabolism levels changed significantly during yak HF cycling. PPAR signaling pathway is the key pathway of lipid metabolism, in which Ppard is a nuclear receptor which acts as sensor of fatty acid and lipid energy metabolism. Retinoic acid (RA) and long-chain fatty acids (LFA) are transported into the nucleus through Crabp2 and Fabp5, respectively. They bind with Ppard to activate PPAR signaling pathway which regulates lipid synthesis and metabolism, and are related to cell survival and cell proliferation [39,40]. Pathway activation affects the expression of downstream related target proteins such as Plin2, Apol, Apoa1, and Apoh. Perilipin 2 (Plin2) is a lipid droplet surface protein that is involved in formation, stability, and trafficking events within the cell (Figure 6A,B) [41]. Apol, Apoa1, and Apoh are members of the apolipoprotein family and may play a role in lipid exchange and transportation throughout the body [42,43,44]. In the enrichment analysis of DEPs, Crabp2, Fabp5, and Plin2 are up-regulated in catagen and down-regulated in anagen. Apoa1 and Apoh were down-regulated in catagen and up-regulated in anagen (Figure 6A,B). In general, yak skin at the catagen and anagen stages contains more lipids while the accumulation of lipids in the skin occurs during anagen. This is highly consistent with the metabolic changes of yak body, which stores fat in autumn and winter against cold, and in summer, the body gradually accumulates fat due to abundant water and grass, which may be the result of a long-term evolutionary adaptation of the yak to the harsh environment it inhabits. The relationship between lipid metabolism in dermis and HF cycle has been an important topic of research [45,46]. Previous studies revealed that intradermal adipocyte lineage cells are necessary and sufficient to drive follicular stem cell activation [45]. At the onset of new hair growth, hair follicles secrete activators of adipogenesis, while at the end of hair growth, a reduction in the secretion of activators or potentially an increase in the secretion of inhibitors of adipogenesis, results in fat lipolysis [46]. Sfrp1 is an antagonist of WNT signaling while on the other hand it is a known adipokine [47]. WNT signaling also targets Ppard with the expression sites of Sfrp1 and Ppard in yak hair follicles being basically the same. Notably, Sfrp1 is highly expressed in L-anagen and catagen, with low expression in other stages. In contrast, Ppard is highly expressed in E-anagen and M-anagen and low in catagen. Their expression trends are opposite, and especially in the hair DP. This indicates that WNT and PPAR signaling pathways may participate in the yak HF cycling. How the above proteins and transcription factors regulate yak HF cycling in space and time still needs to be specifically studied.

## 4. Materials and Methods

### 4.1. Animal and Sample Collection

In this study, samples were collected from five healthy male yaks aged two years in the pastoral area of Tianzhu County. The back skin samples were collected in January, April, June, September, and November within the same year. The samples at different time points came from the same yak to avoid biological individual differences. The samples were collected under local anesthesia. Skin samples of 1 cm × 2 cm size were cut, washed with normal saline, dried with absorbent paper, and the samples divided into two parts for tissue sectioning and proteomics. The animal care and experimental protocols were approved by the Animal Ethics Committee of Gansu Agricultural University and conducted according to the Animal Ethics Regulations of the People’s Republic of China.

### 4.2. Preparation of Tissue Sections and HE Staining

Skin samples during the different stages of HF were stored in a 4% paraformaldehyde solution, softened, dehydrated, embedded in paraffin, sectioned at a thickness of 5 μm using a microtome and then deparaffinized. The sections were routinely dewaxed and stained using hematoxylin and eosin (H.E) [6].

### 4.3. Protein Extraction and SDS-PAGE

An amount of 50 mg yak skin sample was added to lysis buffer (8M urea, Amresco, Solon, OH, USA), swirled and thoroughly mixed, (lysate: protease inhibitor 50:1 Add protease inhibitor) ultrasound for 1 s, stop for 1 s, and cycled for a total of 120 s. After centrifuging at 14,000× *g* for 20 min, the supernatant was separated and left for 10 min to quantify, and stored in a freezer at −80 °C. The concentration of extracted protein was determined using the Bradford method. Briefly, the sample was diluted with lysis buffer so that its final concentration falls within the standard curve range. An amount of 10 μL each of the diluted samples and standard products (the BSA dissolved into the lysis buffer to a series of standard protein concentrations) were taken and mixed with 300 μL protein quantification dye in the dark for 10 min. The absorbance of standard and sample was measured simultaneously at 595 nm with a microplate reader (DR200B, Waltham, MA, USA) followed by drawing a standard curve according to the relationship between absorbance and concentration of each tube of standard. This was then used to calculate the sample’s concentration. After that, 5 μg of each sample was taken for SDS-PAGE electrophoresis (Bio-rad, Hercules, CA, USA), Coomassie brilliant blue staining for 30 min and decolorization until the background was clear [48].

### 4.4. Protein Sample Digestion and TMT Labelling

We used 1× TEAB (Sigma-Aldrich, St. Louis, MO, USA) to adjust the protein concentration to 0.5 μg/μL. An amount of 200 μg proteins was taken and added to a final concentration of 10 mM TCEP (Sigma-Aldrich, USA) and 25 mM CAA (Sigma-Aldrich, USA) at 37 °C for 30 min to react. Pre-washed beads with water were added and incubated at room temperature for 18 min. After incubation, the supernatant was discarded, 40 μL 1× TEAB was added to re-suspend the washed beads, and trypsin (Promega, Madison, WI, USA) was added at a protein-to-trypsin ratio of 10:1 for digestion at 37 °C for more than 4 h. After that, 5% TFA (Amresco, USA) was added to terminate enzymatic cleavage and then freeze dried. The TMT reagent (Thermofisher, Waltham, MA, USA) was thawed at room temperature, and then 41 μL of acetonitrile (J.T. Baker, Phillipsburg, NJ, USA) was added. The mixture was shaken for 5 min and centrifuged. It was added to 100 μg of the digested sample and reacted at room temperature for 1 h. Ammonia water (Wako Pure Chemical Industries Ltd., Richmond, VA, USA) was added to terminate the reaction. The samples after labeling were mixed, vortexed and centrifuged before freeze drying via vacuum centrifugation. The labeled samples after mixing were dissolved in 100 μL of mobile phase A (100% water, 0.1% formic acid (Sigma-Aldrich, USA) and centrifuged at 14,000× *g* for 20 min. The supernatant was then fractionated using high-performance liquid chromatography (HPLC) [49].

### 4.5. LC–MS/MS Analysis and Database Search

The resulting LC-MS/MS data were processed using the Thermo Proteome Discoverer (v.2.4). Tandem mass spectra (Rigol L-3000, China) were searched against Bos taurus (37,513 sequences) from the UniProt database (http://beta.uniprot.org/, accessed on 25 May 2021). Trypsin was specified as a cleavage enzyme, allowing up to two missing cleavages. The mass tolerance for precursor ions was set as 15 ppm in the first search and 5 ppm in the main search, and the mass tolerance for fragment ions was set as 0.02 Da. Carbamidomethyl on Cys was specified as a static modification, and oxidation on Met was specified as a dynamic modification. The false discovery rate (FDR) was adjusted to <1.0%, and the minimum score for peptides was set as >40 [50].

### 4.6. Bioinformatics Analysis

Gene Ontology (GO) annotation of the proteome was derived from the UniProt-GOA database (http://www.ebi.ac.uk/GOA/, accessed on 25 May 2021) that classified proteins into three categories: biological process (BP), cellular compartment (CC), and molecular function (MF). The Eukaryotic Orthologous Groups (KOG) database was used for the functional classification of differentially expressed proteins (DEPs), FC (Fold change) >1.2. The Kyoto Encyclopedia of Genes and Genomes (KEGG) database was used to annotate metabolic pathways. For GO and KEGG enrichment analyses, since the repetition times of the sample are greater than or equal to 3 times, *t*-test was directly used for DEPs analysis and a corrected *p*-value of <0.05 was considered significant. Interactions between proteins were evaluated with the STRING database (http://string-db.org/, accessed on 25 May 2021) based on a minimum interaction score cut-off of 0.400. Predicted interactions in this database are based on either direct or indirect evidence from prior studies, co-expression analyses, and genomic/high-throughput analyses. The software used for figures are Graphpad prism (10.1.2) and Cytoscape (3.9.1).

### 4.7. Western Blot Analysis

Based on existing protocols from the literature, appropriate volumes of RIPA buffer (Solarbio, Beijing, China) together with PMSF were used to extract total protein from skin tissues. After centrifugation, the supernatant containing total protein was collected and denatured with 4× SDS-PAGE loading buffer (Solarbio, Beijing, China). The denatured proteins were electrophoresed using SDS-PAGE (Bio-Rad, Irvine, CA, USA) and transferred to PVDF membranes (Millipore, Darmstadt, Germany). The membrane was blocked for 2 h at room temperature in PBST solution, containing 5% skim milk powder. It was then incubated with rabbit anti-SFRP1 antibody (1:2000, Proteintech, Wuhan, Hubei, China), rabbit anti-ppard antibody (PPARD, 1:1000; Affinity, Beijing, China) and mouse anti-β-actin antibody (1:2500; Bioss, Beijing, China) at 4 °C overnight. Corresponding secondary antibodies were used to incubate membranes for 1 h at room temperature after the primary antibody was washed off with PBST. Finally, ECL detection kit (Beyotime, Shanghai, China) was used for color reaction, and Amersham Imager 600 (GE Healthcare Life Sciences, Marlborough, MA, USA) was used to observe the resulting bands. β-actin was used as the control and the results were analyzed using Image J software v. 1.38 at https://imagej.nih.gov/ij/, accessed on 4 August 2022 (National Institutes of Health, Bethesda, MD, USA)

### 4.8. Immunohistochemistry

The prepared paraffin sections (5 μm) of tissues were deparaffinized using xylene and hydrated by gradient alcohol. After washing the sections in water, antigen retrieval was performed using sodium citrate buffer (pH = 6.0) with a microwave. The endogenous horseradish peroxidase activity was inhibited with an endogenous pertoxidase blocker at 37 °C. After blocking with 5% BSA at 37 °C for 1 h, sections were incubated with rabbit anti-SFRP1 antibody (1:200, Proteintech, China), rabbit anti-ppard antibody (PPARD, 1:200; Affinity, China), diluted in 5% BSA at 4 °C overnight, respectively. The sections were then incubated with secondary antibodies (1:2000; Bioss, Beijing, China) at 37 °C for 1 h, respectively. Finally, the signals were visualized with the DAB Kit (ZLI 9018; Zhongshan Golden Bridge, Beijing, China). The sections were counterstained with haematoxylin. The staining showed strong expression in brown, medium expression in yellow, weak expression in light yellow and no expression in non-staining.

### 4.9. Statistical Analysis

All data are presented as mean ± standard deviation (SD) of independent experiments. Statistical analyses were performed using SPSS 22.0 software (IBM, Armonk, NY, USA). Comparisons were conducted using one-way analysis of variance (ANOVA), and *p* < 0.05 was regarded as statistically significant.

## 5. Conclusions

This study showed that the protein regulatory network of the yak HF growth cycle is complex and dynamically changing and revealed key candidate proteins that may affect yak hair follicle development. These findings provided detailed data for further understanding the plateau adaptation mechanism of the yak, which is of great significance to make better use of the yak livestock resources and enhance their economic value.

## Data Availability

All data generated during the current study is included in this manuscript.

## Supplementary Materials

The following supporting information can be downloaded at: https://www.mdpi.com/article/10.3390/ijms26041532/s1.
