# Supplementary figures and images for: Insights from Tandem Mass Tag (TMT) Proteomic Analysis on Protein Network Modification in Control of Yak Hair Follicle Cycle
Source: Int J Mol Sci. 2025 Feb 12;26(4):1532. doi: 10.3390/ijms26041532 (PMC11855600; doi:10.3390/ijms26041532)

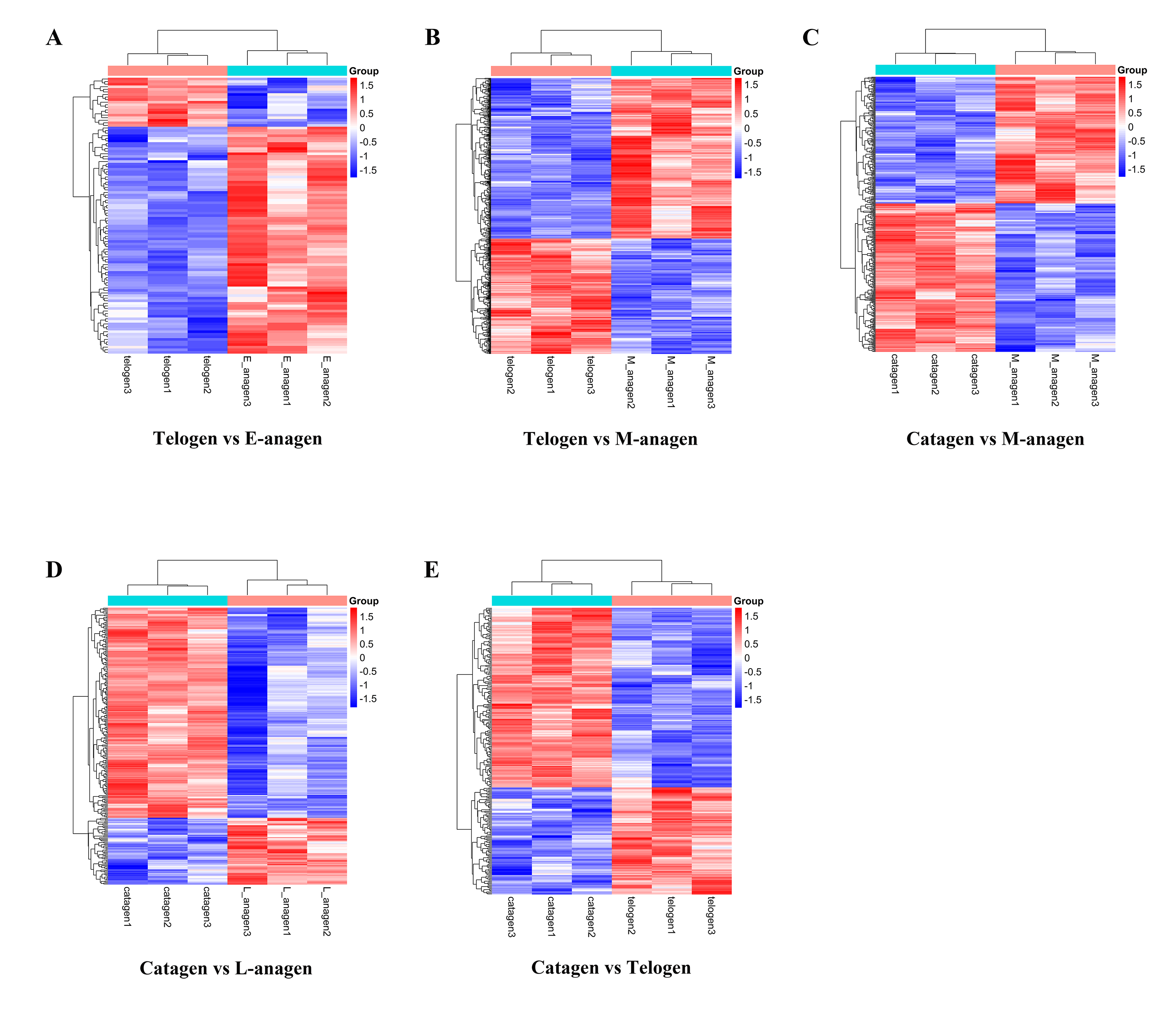

Supplement: Supplementary file 1 [file ijms-26-01532-s001.zip › Figure S1 Heat map of the DEPs between each stage of yak hair cycle..tif]

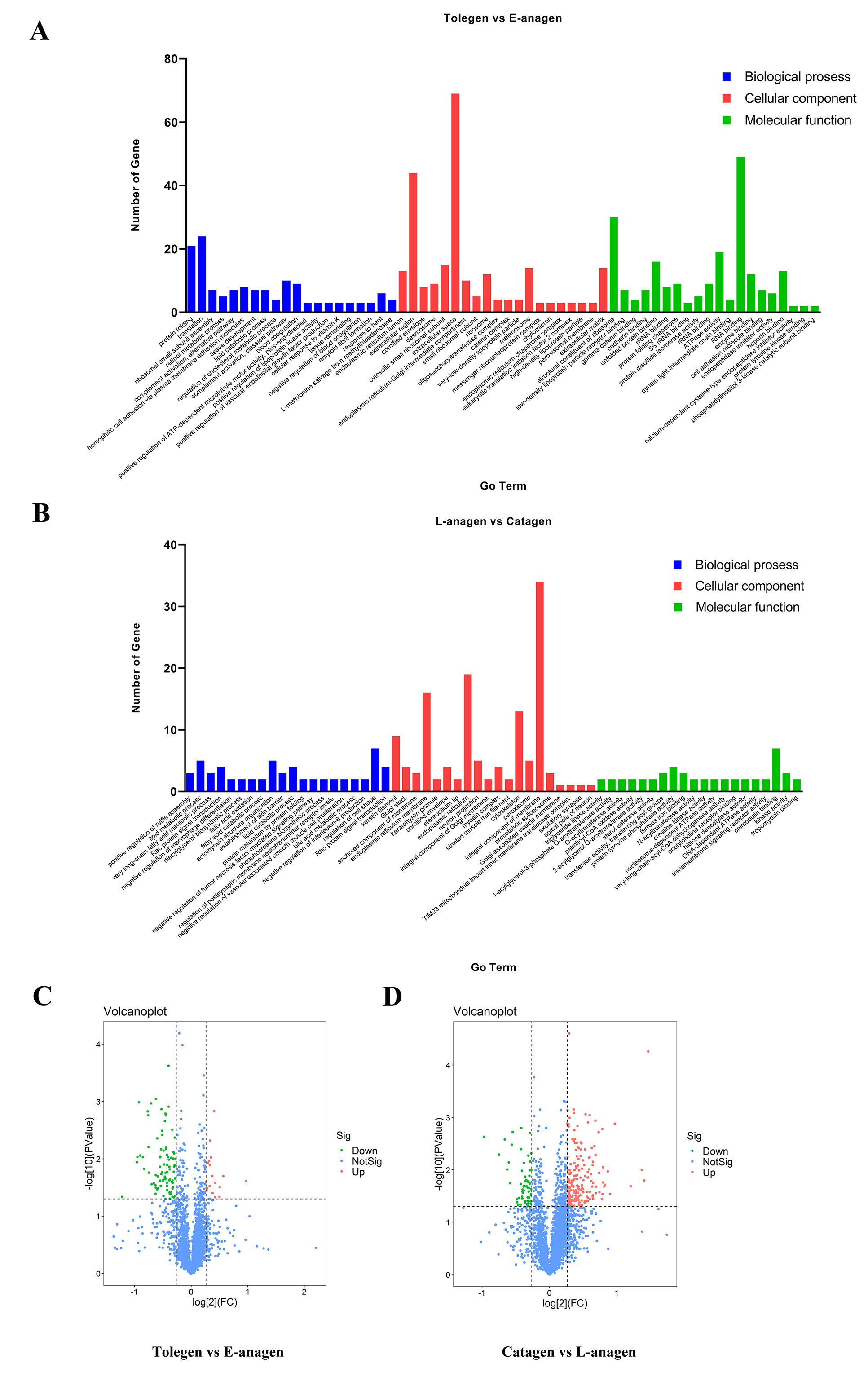

Supplement: Supplementary file 1 [file ijms-26-01532-s001.zip › Figure S2 GO enrichment analyses and Volcano plot of DEPs at the time point of yak hair growth and degeneration.tif]
